# Risk of Major Adverse Cardiovascular Events in Home Dialysis Compared With In-Center Hemodialysis

**DOI:** 10.2215/CJN.0000000579

**Published:** 2024-11-19

**Authors:** Wisam Bitar, Jaakko Helve, Mikko Haapio, Virpi Rauta, Eero Honkanen, Patrik Finne

**Affiliations:** 1 Nephrology Department, University of Helsinki and Helsinki University Hospital, Helsinki, Finland; 2 Finnish Registry for Kidney Diseases, Finnish Kidney and Liver Association, Helsinki, Finland; 3 Strategy and Development, Helsinki and Uusimaa Hospitals District, Helsinki, Finland

**Keywords:** cardiovascular events, chronic dialysis, hemodialysis, peritoneal dialysis

## Abstract

**Key Points:**

- We observed a comparable cumulative incidence of major adverse cardiovascular event (MACE) in in-center hemodialysis (IC-HD) and continuous ambulatory peritoneal dialysis (PD) patients, which was higher than in automated PD and home hemodialysis patients.
- After adjustment for confounders, there was no difference in risk of MACE between patients on home dialysis modalities and IC-HD.
- Compared with IC-HD, PD was associated with lower risk of MACE among female patients and higher risk among male patients.

**Background:**

Among dialysis patients, cardiovascular events are the leading cause of death. Little is known about how the frequency and type of cardiovascular events differ between various dialysis modalities. We compared risk of major adverse cardiovascular events (MACEs) in patients who started continuous ambulatory peritoneal dialysis (CAPD), automated peritoneal dialysis (APD), and home hemodialysis with in-center hemodialysis (IC-HD) patients.

**Methods:**

We included 968 patients who entered dialysis in the Helsinki-Uusimaa health care district in Finland from 2004 to 2017, of whom 162 were on CAPD, 229 on APD, 145 on home hemodialysis, and 432 on IC-HD at day 90 from the start of dialysis. MACE was defined as acute myocardial infarction, stroke, or death due to cardiovascular disease. The cumulative incidence of the first MACE was calculated. Cox regression was used to compare risk of MACE between dialysis modalities with adjustment for potential confounding factors.

**Results:**

Of all 968 patients, 195 (20%) experienced a MACE during the entire follow-up and 62 (6%) during the first year of follow-up. The cumulative incidence of first MACE was similar in IC-HD and CAPD patients and higher than that in APD and home hemodialysis patients. After adjustment for possible confounders, the hazard ratio (HR) of MACE was 1.22 (95% confidence intervals [CIs], 0.73 to 2.05) for CAPD, 0.86 (95% CI, 0.47 to 1.57) for APD and 0.67 (95% CI, 0.30 to 1.50) for home hemodialysis compared with IC-HD. Unexpectedly, compared with IC-HD, peritoneal dialysis associated with lower risk of MACE among female patients (HR, 0.37; 95% CI, 0.14 to 0.99) and higher risk among male patients (HR, 1.80; 95% CI, 1.11 to 2.92).

**Conclusions:**

In this cohort, the risk of MACE was comparable across in-center and home dialysis modalities. However, the result differed between male patients and female patients, which requires further research.

## Visual Abstract

**Figure:**
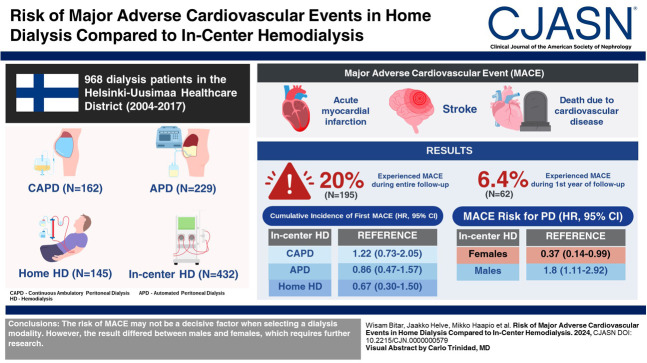

## Introduction

Cardiovascular disease is the most common cause of death among dialysis patients and causes approximately 40% of the deaths.^1^ Dialysis patients have been shown to have a 10-fold to 20-fold risk of dying from cardiovascular diseases compared with general population.^2,3^

The increased cardiovascular mortality observed in the dialysis population could be explained in part by factors related to uremia such as oxidative stress and inflammation.^4^ Compared with hemodialysis, peritoneal dialysis (PD) provides more stable removal of uremic products and fluid, contributing to reduced hemodynamic stress. This could theoretically contribute to lower risk of cardiovascular events. In addition, the longer preservation of residual kidney function observed in PD^5^ has been connected to improved cardiac parameters^6,7^ and reduction in cardiovascular events and mortality.^8,9^ On the other hand, PD may increase cardiovascular risk by causing hyperglycemia, obesity, or dyslipidemia.

Home hemodialysis typically allows more frequent dialysis than three times a week in-center hemodialysis (IC-HD). Patients on more frequent hemodialysis have been shown to have better BP control, left ventricular mass reduction, and less often intradialytic hypotension than patients on thrice-weekly IC-HD.^10,11^ Risk of cardiac death has been observed to vary from day to day among thrice-weekly IC-HD patients, but not among home hemodialysis and PD patients.^12,13^

Several studies have compared mortality and morbidity due to cardiovascular disease between PD and IC-HD patients, but results have been conflicting. In addition, some studies have shown lower risk of cardiovascular related hospitalization or death in frequent home hemodialysis compared with PD and also compared with IC-HD.^14–16^ Compared with IC-HD, PD has been associated with higher risk of emergency hospitalization and mortality due to cardiovascular disease,^17^ but on the other hand, PD has also been associated with lower incidence of congestive heart failure.^18^ A large population-based study showed that PD was noninferior to hemodialysis with regard to cardiovascular mortality.^19^ Notably, all studies have been observational, and the results may have been affected by differences in patient selection to the dialysis modalities and patient characteristics. This underlines the importance of comprehensive adjustment for confounding factors.

We have earlier shown that patients entering automated PD (APD) have similar survival compared with those who enter home hemodialysis.^20^ However, it is not well understood how these dialysis modalities affect patients' risk of cardiovascular events. To investigate this, we collected, in addition to data on dialysis, comprehensive information on various other factors possibly affecting cardiovascular risk. This enabled thorough adjustment for potential confounders when comparing the risk of major adverse cardiovascular event (MACE) in patients on home hemodialysis, APD, continuous ambulatory PD (CAPD), and IC-HD.

## Methods

### Study Design and Population

All patients aged 18 years or older who were on one of the home dialysis modalities at day 90 from the beginning of the dialysis therapy in the Helsinki-Uusimaa health care district in Finland were included (*N*=536). In addition, we used Microsoft Excel (the random command) to select a random sample of patients who were on IC-HD at day 90 from starting dialysis (*N*=432) to represent the whole group of IC-HD patients (*N*=1211) (Supplemental Figure 1). The reason for selecting a random sample was that the number of IC-HD patients was much larger than that of home dialysis patients, and all data were collected manually from the patient files. All study patients (*N*=968) had started their dialysis therapy between 2004 and 2017 (Supplemental Table 1).

### Data Collection

Data were extracted retrospectively from the patient data system of Helsinki University Hospital into a structural database. The last available data before start of dialysis were collected on cardiovascular and other comorbidities, primary kidney disease, electrocardiogram and echocardiography parameters, BP, and laboratory tests. A summary of the data are displayed in Supplemental Table 2. MACE was defined as myocardial infarction, ischemic or hemorrhagic cerebrovascular stroke, or death due to cardiovascular causes.

### Statistical Analyses

**Figure 1:**
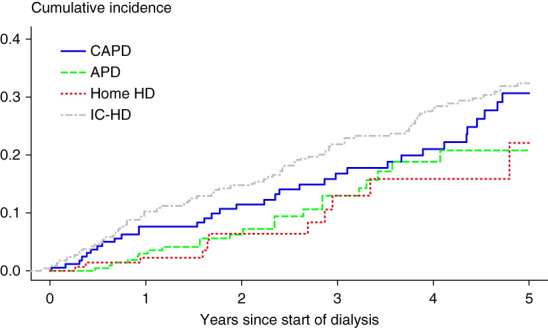
**Cumulative incidence of the first MACE according to dialysis modality.** APD, automated peritoneal dialysis; CAPD, continuous ambulatory peritoneal dialysis; HD, hemodialysis; IC-HD, in-center hemodialysis; MACE, major adverse cardiovascular event; PD, peritoneal dialysis.

The cumulative incidence of first MACE was estimated considering death due to noncardiac causes as a competing risk event according to dialysis modality (CAPD, APD, home hemodialysis, and IC-HD) at day 90 from the beginning of dialysis (Figure 1).

We used Cox regression to assess hazard ratios (HRs) of first MACE associated with the dialysis treatment modality at 90 days from start of dialysis. Patients were censored at kidney transplantation (*N*=444), 5 years from starting dialysis (*N*=141), recovery of kidney function (*N*=7), loss of follow-up (FU) (*N*=3), and end of the year 2019 (*N*=92) or death due to noncardiac causes (*N*=151).

Missing values were imputed using predicting variables as appears in Supplemental Table 2. The multiple imputation tool in SPSS was used to impute one dataset with complete information for all analyzed variables. To adjust for confounding, we developed propensity scores using binary logistic regression for CAPD versus IC-HD, APD versus IC-HD and home hemodialysis versus IC-HD pairwise. All explanatory variables for the propensity scores were selected by a stepwise forward procedure. Supplemental Table 2 displays the related variables.

The incidence rate and incidence rate ratios (IRRs) of MACE were assessed for CAPD, APD, home hemodialysis, and IC-HD patients. This analysis included also subsequent events, not only the first MACE of each patient. Confidence intervals (CIs) of IRR were calculated.^21^ Sensitivity analyses were conducted to ensure that the main result did not change by alternative analytic strategies. These included doing separate analyses for male patients and female patients and for patients with or without cardiovascular comorbidities before start of dialysis.

The cumulative incidence was estimated using the R Software (version Ri386 3.6.0, the cmprsk package, cuminc function), while the other statistical analyses were performed using SPSS (version 25).

## Results

### Population Characteristics

A total of 968 patients were included in the study cohort. At day 90 from the beginning of dialysis, 162 were on CAPD, 229 on APD, 145 on home hemodialysis, and 432 on IC-hemodialysis. Patients on CAPD and IC-hemodialysis were older and had more often cardiovascular comorbidities before dialysis start compared with patients on APD or home hemodialysis (Table 1). IC-HD patients more often had diabetes and less often GN or polycystic kidney degeneration compared with home dialysis patients. More detailed patient characteristics are shown in Supplemental Table 2.

**Table 1:** Baseline patient characteristics according to dialysis modality

Dialysis Modality^a^	CAPD	APD	Home Hemodialysis	IC-HD	*P* Value		
					CAPD versus IC-HD	APD versus IC-HD	Home Hemodialysis versus IC-HD
Number of patients	162	229	145	432			
Male sex, %	70	66	68	64	0.17	0.60	0.39
Age, yr, median (IQR)	65 (52–74)	50 (40–61)	50 (42–60)	66 (56–73)	0.50	<0.001	<0.001
**Primary kidney disease, %**					0.003	<0.001	<0.001
GN	17	22	24	8	^b^	^b^	^b^
Polycystic kidney disease	6	15	31	8		^b^	^b^
Diabetes	19	21	17	33			^b^
Others	11	12	9	51	^b^	^b^	^b^
**A history of at least one comorbidity, %**	29	13	13	32	0.56	<0.001	<0.001
Myocardial infarction	11	5	6	13	0.64	0.003	0.04
Cerebrovascular infarction	1.2	0.4	1.4	1.4	0.88	0.26	0.99
Cerebrovascular bleeding	0.6	0.0	0.0	0.9	0.71	0.14	0.25
FU-time, yr, median (IQR)	2.7 (1.4–4.4)	1.9 (1.1–3.6)	1.6 (0.88–3.0)	2.6 (1.4–4.4)	0.60	0.001	<0.001
At least one MACE event, %	21	9.6	7.6	24	0.40	<0.001	<0.001
Deaths in 5 yr^c^, *N*	63	25	10	174	0.76	<0.001	<0.001
Cardiovascular deaths^c^, *N*	22	10	5	70	0.43	<0.001	<0.001

APD, automated peritoneal dialysis; CAPD, continuous ambulatory peritoneal dialysis; FU, follow-up; IC-HD, in-center hemodialysis; IQR, interquartile range; MACE, major adverse cardiovascular event; *N*, number; PD, peritoneal dialysis; %, percentage.

a Dialysis modality at 90 days from start of KRT.

b Points with statistically significant differences in the primary kidney disease group.

c During follow-up period.

### Cumulative Incidence and HR of the First MACE

Of the 968 patients, 172 (18%) experienced at least one MACE event during the FU. The cumulative incidence of a first MACE was 8% for CAPD, 3% for APD, 2% for home hemodialysis, and 10% for IC-HD patients at 1 year from the beginning of dialysis (Figure 1). In unadjusted and in age-adjusted and sex-adjusted analyses, APD and home hemodialysis patients had a lower risk of a first MACE compared with IC-HD patients (Table 2). However, after adjustment for propensity scores, these differences diminished and were no longer statistically significant, HR 0.86 (95% CI, 0.47 to 1.57) for APD and HR 0.67 (95% CI, 0.30 to 1.50) for home hemodialysis compared with IC-HD. When comparing CAPD with IC-HD, there was no statistically significant difference between hazards of first MACE.

**Table 2:** Hazard ratios of first major adverse cardiovascular event according to dialysis modality during 5 years from the start of KRT

Analysis	HR of First MACE	95% CI of HR	
		Lower	Upper
**HR of first MACE, unadjusted**			
IC-HD (reference)			
*CAPD*	0.83	0.56	1.22
*APD*	0.49	0.31	0.77
*Home hemodialysis*	0.42	0.23	0.78
**HR of first MACE, adjusted for age and sex**			
IC-HD (reference)			
*CAPD*	0.81	0.55	1.20
*APD*	0.62	0.38	1.00
*Home hemodialysis*	0.53	0.28	1.00
**HR of first MACE, adjusted for propensity score**			
CAPD (compared with IC-HD)	1.22	0.73	2.05
APD (compared with IC-HD)	0.86	0.47	1.57
Home hemodialysis (compared with IC-HD)	0.67	0.30	1.50

APD, automated peritoneal dialysis; CAPD, continuous ambulatory peritoneal dialysis; CI, confidence interval; HR, hazard ratio; IC-HD, in-center hemodialysis; MACE, major adverse cardiovascular event; PD, peritoneal dialysis.

### IR of MACE

By calculating the IR, we considered all MACE events, not only the first MACE event of each patient. The IR was 109 MACE per 1000 patient-years for CAPD patients while it was 50 for APD, 32 for home hemodialysis, and 91 for IC-HD patients (Table 3). The corresponding IRR was 1.20 (95% CI, 0.99 to 1.46) for CAPD, 0.55 (95% CI, 0.45 to 0.67) for APD, and 0.35 (95% CI, 0.29 to 0.43) for home hemodialysis compared with IC-HD patients. Death due to cardiovascular disease comprised 52% of all MACE events during the study FU period, followed by myocardial infarction (27%) and cerebrovascular infarction (17%) and cerebrovascular bleeding (4%) (Supplemental Table 3).

**Table 3:** Incidence rate and incidence rate ratio of major adverse cardiovascular event

Dialysis Modality	CAPD	APD	Home Hemodialysis	IC-HD
Number of PY	375	522	345	1266
Number of MACE events	41	26	11	115
IR (per 1000 PY) of MACE	109	50	32	91
IRR of MACE (95% CI)	1.20 (0.99–1.46)	0.55 (0.45–0.67)	0.35 (0.29–0.43)	1 (reference)

APD, automated peritoneal dialysis; CAPD, continuous ambulatory peritoneal dialysis; CI, confidence interval; IC-HD, in-center hemodialysis; IR, incidence rate per 1000 patient-years; IRR, incidence rate ratio; MACE, major adverse cardiovascular events; PD, peritoneal dialysis; PY, patient-years.

### Sensitivity Analyses

As a sensitivity analysis, we assessed the risk of getting the first MACE separately in patients with and without cardiovascular comorbidities at the time of dialysis initiation. With propensity score adjustment, the results were similar to those shown in Table 2, and there were no significant interactions between cardiovascular comorbidities and treatment modalities. For patients without prior cardiovascular comorbidities and compared with IC-HD, the HR for MACE was 0.97 (95% CI, 0.44 to 2.13) for CAPD, 0.97 (0.44 to 2.15) for APD, and 1.00 (0.37 to 2.72) for home hemodialysis.

When analyzed separately by sex, and with adjustment for propensity scores, the HR of MACE was 1.80 (95% CI, 1.11 to 2.92) for male patients and 0.37 (0.14 to 0.99) for female patients among those who started PD compared with IC-HD. The interaction between sex and treatment modality was statistically significant (*P* = 0.006). We also analyzed separately CAPD and APD: The difference between sexes remained similar in both PD modalities, thus PD associating with a higher risk of MACE among male patients and a lower risk among female patients (Supplemental Table 4). We further compared PD, CAPD, and APD with home hemodialysis separately by sex, and the result was similar to the comparison with IC-HD. We also analyzed separately patients who had or had not a history of cardiovascular comorbidities before start of dialysis, and again the difference between sexes was observed in both groups: compared with IC-HD, PD associated with higher risk of MACE among male patients and lower risk among female patients as compared with IC-HD.

In the main analysis, we did not censor at time of dialysis modality change. Of the patients who were on PD at day 90, 24% had transferred to IC-HD before the end FU, and of the patients who were on IC-HD, 5% transferred to PD and 5% to home hemodialysis. We performed a sensitivity analysis, with censoring also at the time of dialysis modality change (PD, home hemodialysis, or IC-HD). The results were comparable with the main analysis (Supplemental Table 5).

## Discussion

We observed that patients who started APD or home hemodialysis had lower risk of MACE compared with those who started IC-HD, but after adjustment for confounding factors, the difference was no longer significant. Patients on APD and home hemodialysis showed similar characteristics, and they were younger and had fewer comorbidities than patients on CAPD and IC-HD. During the 5-year FU, 18% of the patients experienced at least one MACE, cardiovascular deaths comprising half of them.

Several earlier studies have compared risk of cardiovascular events and mortality between dialysis modalities. Hu *et al.* showed that PD was not inferior to IC-HD regarding cardiovascular events.^19^ The authors used propensity score matching with comorbidities but recommended to include more information on confounding factors in future studies. For example, their study did not have detailed data on cardiovascular comorbidities, laboratory results, electrocardiogram, and cardiac ultrasound findings, which all were available in our study. A meta-analysis published in 2019 based on five studies^22–26^ compared cardiovascular adverse events between patients who started PD with those who started hemodialysis,^27^ showing no difference between the treatment modalities. However, one of the included studies showed a lower risk of cardiovascular events among PD compared with hemodialysis patients.^22^ Notably, only one of the included studies^23^ contained data on both laboratory values and prior cardiovascular comorbidities. Some studies have shown lower risk of hospitalization for cardiovascular events among patients on frequent home dialysis compared with PD patients.^14–16^ Compared with IC-HD, PD has been connected to higher risk of emergency hospitalization for cardiovascular disease^17^ and lower risk of hospitalization for congestive heart failure.^18^ We did not have data on hospitalizations, but we analyzed the risk of cardiovascular death, which was in line with that of MACE.

Unexpectedly, we observed that among females, PD associated with a clearly lower risk of MACE compared with IC-HD or home hemodialysis, while the association was the opposite among males. Earlier studies have not analyzed separately for male patients and female patients how various dialysis modalities associate with risk of MACE. Hu *et al.* observed a higher HR of death for PD compared with hemodialysis among female patients than male patients but did not present results on cardiovascular events by sex.^19^ The reason for the different finding between male patients and female patients in our study remains unclear. We also found a similar tendency, but statistically nonsignificant, for risk of cardiovascular death. Regarding all-cause death, we observed no difference between sexes when comparing PD and hemodialysis. Based on our findings, we suggest that future studies compare outcomes of PD and hemodialysis separately by sex.

There are many strengths in our study. As a population-based study, it included all patients in Helsinki-Uusimaa health care district who were on home dialysis at day 90 and a randomly selected sample of IC-HD patients. Of all patients who entered KRT, the proportion of home dialysis patients was relatively high. As a result, these patients were not highly selected, and this may have reduced selection bias. Furthermore, we had complete data on MACE and comprehensive data on possible confounders. For most variables, <10% of data were missing, but for some variables, the level of missingness was higher. Statistical imputation was used to replace missing values, to include all patients and avoid selection bias. Notably, our study is observational, and despite adjusting for a large number of confounders, the results may still be affected by residual confounding. For example, we did not have information on fluid status or residual kidney function. As the numbers of patients and cardiovascular events were rather low, our study does not have the statistical power to show small differences between the dialysis modalities. In addition, the results may not be generalized to other settings in which, for instance, different selection criteria are applied for patients to receive home dialysis. Thus, it is important that these results are validated in other datasets in the future.

Regarding choosing between dialysis modalities, many nephrological centers have a PD first or home first policy, and the Finnish Society of Nephrology has published a strategy for increasing the proportion of home dialysis in Finland.^28^ When deciding the dialysis modality, it is important that the patient is involved and well-informed. An important piece of information is how the dialysis modality affects various aspects of the patient's outcome. We have earlier shown that APD, and home hemodialysis are similar, and not statistically different than CAPD, with regard to survival. Our study does not demonstrate significant differences in risk of MACE between various dialysis modalities, which is important information for both health care professionals and patients when selecting the most appropriate dialysis modality. Notably, we found a difference between the sexes, so that PD, as compared with IC-HD, was connected to higher risk of MACE among male patients than female patients. This finding needs to be evaluated in further studies.

In conclusion, the risk of MACE was comparable across in-center and home dialysis modalities in this cohort study suggesting that the risk of cardiovascular events may not be a decisive factor when selecting a particular dialysis modality. Unexpectedly, we observed that PD is associated with lower risk of MACE among female patients and higher risk among male patients compared with IC-HD. Further research is needed to evaluate these findings and explore additional clinical outcomes associated with different dialysis modalities to guide informed decision-making in clinical practice.

## Supplementary Material

## Data Availability

Data cannot be shared. The data protection law of Finland (December 5, 2018/1050), which is based on the General Data Protection Regulation of European Union, does not allow public (or any) sharing of individual patients' data. Our research permission granted by Helsinki University Hospital does not allow sharing of individual patients' data. Researchers can submit a research application to the Helsinki University Hospital in order to get access to the data.

## Supplemental Material

Supplemental Figure 1. Study cohort according to dialysis modality at day 90 from starting dialysis treatment.

Supplemental Table 1. Yearly distribution of patients according to dialysis modality at day 90 from starting dialysis treatment over the study period.

Supplemental Table 2. Detailed characteristics of the study population.

Supplemental Table 3. Distribution of MACE according to dialysis modality.

Supplemental Table 4. Comparison of HRs of MACE between male patients and female patients with propensity score adjustment.

Supplemental Table 5. HRs of the first MACE according to dialysis modality during 5 years from the start of KRT, with censoring at change of dialysis modality, kidney transplantation, loss of FU, recovery of kidney function, noncardiac death, and end of FU.
