# Supplementary material for: Risk of Major Adverse Cardiovascular Events in Home Dialysis Compared With In-Center Hemodialysis
Source: Clin J Am Soc Nephrol. 2024 Nov 19;20(1):81–7. doi: 10.2215/CJN.0000000579 (PMC11737445; doi:10.2215/CJN.0000000579)
Supplement: Supplementary file 1 [file cjasn-20-081-s001.pdf]

ASN American Society of Nephrology | JASN Journal of the American Society of Nephrology | KIDNEY360 Accessing Our World From Every Angle | CJASN Clinical Journal of the American Society of Nephrology

ASN Journal Disclosure Form

As per ASN journal policy, I have disclosed any financial relationships or commitments I have held in the past 36 months as included below. I have listed my Current Employer below to indicate there is a relationship requiring disclosure. If no relationship exists, my Current Employer is not listed.

W. Bitar has nothing to disclose.

I understand that the information above will be published within the journal article, if accepted, and that failure to comply and/or to accurately and completely report the potential financial conflicts of interest could lead to the following: 1) Prior to publication, article rejection, or 2) Post-publication, sanctions ranging from, but not limited to, issuing a correction, reporting the inaccurate information to the authors' institution, banning authors from submitting work to ASN journals for varying lengths of time, and/or retraction of the published work.

Name: Wisam Bitar
Manuscript ID: CJASN-2024-000586
Manuscript Title: Risk of Major Adverse Cardiovascular Events in Home Dialysis Compared to In-Center Hemodialysis
Date of Completion: July 16, 2024
Disclosure Updated Date: July 16, 2024

ASN
American Society of Nephrology
JASN
Journal of the American Society of Nephrology
KIDNEY360
Accessing Our World From Every Angle
CJASN
Clinical Journal of the American Society of Nephrology

ASN Journal Disclosure Form

As per ASN journal policy, I have disclosed any financial relationships or commitments I have held in the past 36 months as included below. I have listed my Current Employer below to indicate there is a relationship requiring disclosure. If no relationship exists, my Current Employer is not listed.

P. Finne reports the following:
Employer: Helsinki University Hospital and University of Helsinki; Consultancy: Boehringer Ingelheim; GSK; Astellas; Baxter; AstraZeneca; and Honoraria: Boehringer Ingelheim; GSK; Astellas; Baxter; AstraZeneca.

I understand that the information above will be published within the journal article, if accepted, and that failure to comply and/or to accurately and completely report the potential financial conflicts of interest could lead to the following: 1) Prior to publication, article rejection, or 2) Post-publication, sanctions ranging from, but not limited to, issuing a correction, reporting the inaccurate information to the authors' institution, banning authors from submitting work to ASN journals for varying lengths of time, and/or retraction of the published work.

Name: Patrik Finne
Manuscript ID: CJASN-2024-000586
Manuscript Title: Risk of Major Adverse Cardiovascular Events in Home Dialysis Compared to In-Center Hemodialysis
Date of Completion: July 17, 2024
Disclosure Updated Date: July 17, 2024

ASN
American Society of Nephrology
JASN
Journal of the American Society of Nephrology
KIDNEY360
Accessing Our World From Every Angle
CJASN
Clinical Journal of the American Society of Nephrology

ASN Journal Disclosure Form

As per ASN journal policy, I have disclosed any financial relationships or commitments I have held in the past 36 months as included below. I have listed my Current Employer below to indicate there is a relationship requiring disclosure. If no relationship exists, my Current Employer is not listed.

M. Haapio reports the following:
Consultancy: Sanofi; Honoraria: Sanofi; and Advisory or Leadership Role: Sanofi, Takeda.

I understand that the information above will be published within the journal article, if accepted, and that failure to comply and/or to accurately and completely report the potential financial conflicts of interest could lead to the following: 1) Prior to publication, article rejection, or 2) Post-publication, sanctions ranging from, but not limited to, issuing a correction, reporting the inaccurate information to the authors' institution, banning authors from submitting work to ASN journals for varying lengths of time, and/or retraction of the published work.

Name: Mikko Haapio
Manuscript ID: CJASN-2024-000586
Manuscript Title: Risk of Major Adverse Cardiovascular Events in Home Dialysis Compared to In-Center Hemodialysis
Date of Completion: July 17, 2024
Disclosure Updated Date: July 17, 2024

ASN American Society of Nephrology · JASN Journal of the American Society of Nephrology · KIDNEY360 Accessing Our World From Every Angle · CJASN Clinical Journal of the American Society of Nephrology

ASN Journal Disclosure Form

As per ASN journal policy, I have disclosed any financial relationships or commitments I have held in the past 36 months as included below. I have listed my Current Employer below to indicate there is a relationship requiring disclosure. If no relationship exists, my Current Employer is not listed.

J. Helve reports the following:
Ownership Interest: Orion; and Other Interests or Relationships: Member of the Finnish Society of Nephrology; Administrative director of the Finnish Registry for Kidney Diseases.

I understand that the information above will be published within the journal article, if accepted, and that failure to comply and/or to accurately and completely report the potential financial conflicts of interest could lead to the following: 1) Prior to publication, article rejection, or 2) Post-publication, sanctions ranging from, but not limited to, issuing a correction, reporting the inaccurate information to the authors' institution, banning authors from submitting work to ASN journals for varying lengths of time, and/or retraction of the published work.

Name: Jaakko Helve
Manuscript ID: CJASN-2024-000586
Manuscript Title: Risk of Major Adverse Cardiovascular Events in Home Dialysis Compared to In-Center Hemodialysis
Date of Completion: June 30, 2024
Disclosure Updated Date: June 30, 2024

ASN American Society of Nephrology | JASN Journal of the American Society of Nephrology | KIDNEY360 Accessing Our World From Every Angle | CJASN Clinical Journal of the American Society of Nephrology

ASN Journal Disclosure Form

As per ASN journal policy, I have disclosed any financial relationships or commitments I have held in the past 36 months as included below. I have listed my Current Employer below to indicate there is a relationship requiring disclosure. If no relationship exists, my Current Employer is not listed.

E. Honkanen has nothing to disclose.

I understand that the information above will be published within the journal article, if accepted, and that failure to comply and/or to accurately and completely report the potential financial conflicts of interest could lead to the following: 1) Prior to publication, article rejection, or 2) Post-publication, sanctions ranging from, but not limited to, issuing a correction, reporting the inaccurate information to the authors' institution, banning authors from submitting work to ASN journals for varying lengths of time, and/or retraction of the published work.

Name: Eero Honkanen
Manuscript ID: CJASN-2024-000586
Manuscript Title: "Risk of Major Adverse Cardiovascular Events in Home Dialysis Compared to In-Center Hemodialysis
Date of Completion: June 30, 2024
Disclosure Updated Date: June 30, 2024

ASN Journal Disclosure Form

As per ASN journal policy, I have disclosed any financial relationships or commitments I have held in the past 36 months as included below. I have listed my Current Employer below to indicate there is a relationship requiring disclosure. If no relationship exists, my Current Employer is not listed.

V. Rauta reports the following:
Employer: Coronaria; Consultancy: AstraZeneca; Research Funding: Research funding from Academy of Finland and Business Finland: but these institutions are not-for-profit organizations.; and Honoraria: Baxter, Fresenius, AstraZeneca.

I understand that the information above will be published within the journal article, if accepted, and that failure to comply and/or to accurately and completely report the potential financial conflicts of interest could lead to the following: 1) Prior to publication, article rejection, or 2) Post-publication, sanctions ranging from, but not limited to, issuing a correction, reporting the inaccurate information to the authors' institution, banning authors from submitting work to ASN journals for varying lengths of time, and/or retraction of the published work.

Name: Virpi Rauta
Manuscript ID: CJASN-2024-000586R3
Manuscript Title: Risk of Major Adverse Cardiovascular Events in Home Dialysis Compared to In-Center Hemodialysis
Date of Completion: October 3, 2024
Disclosure Updated Date: October 3, 2024
