# Supplementary material for: Risk of Major Adverse Cardiovascular Events in Home Dialysis Compared With In-Center Hemodialysis
Source: Clin J Am Soc Nephrol. 2024 Nov 19;20(1):81–7. doi: 10.2215/CJN.0000000579 (PMC11737445; doi:10.2215/CJN.0000000579)
Supplement: Supplementary file 2 [file cjasn-20-081-s002.pdf]

# Table of Contents

Supplemental Figure 1 ........................................................................................................ 1

Supplemental Table 1 ........................................................................................................ 2

Supplemental Table 2 ........................................................................................................ 3

Supplemental Table 3 ........................................................................................................ 5

Supplemental Table 4 ........................................................................................................ 6

Supplemental Table 5 ........................................................................................................ 7

# Supplemental Figure 1

*Supplemental Figure 1. Study cohort according to dialysis modality at day 90 from starting Dialysis Treatment.*

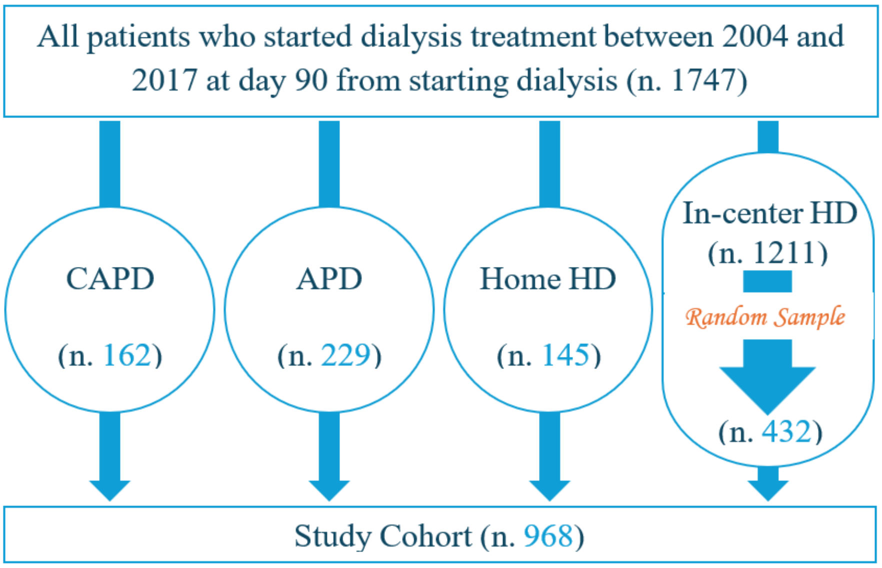


Abbreviations: (n.), patients number; PD, peritoneal dialysis; HD, hemodialysis; CAPD, continuous ambulatory PD; APD, automated PD.

# Supplemental Table 1

**Supplemental Table 1.** Yearly distribution of patients according to dialysis modality at day 90 from starting dialysis treatment over the study period

| Year | PD (n.) | Home HD (n.) | In-center HD (n.) | All Patients (n.) |
|---|---|---|---|---|
| 2004 | 28 | 12 | 28 | 68 |
| 2005 | 29 | 15 | 25 | 69 |
| 2006 | 38 | 3 | 18 | 59 |
| 2007 | 18 | 9 | 30 | 57 |
| 2008 | 36 | 12 | 29 | 77 |
| 2009 | 40 | 8 | 22 | 70 |
| 2010 | 26 | 7 | 27 | 60 |
| 2011 | 29 | 12 | 23 | 64 |
| 2012 | 20 | 9 | 41 | 70 |
| 2013 | 37 | 14 | 26 | 77 |
| 2014 | 27 | 11 | 39 | 77 |
| 2015 | 20 | 13 | 36 | 69 |
| 2016 | 26 | 11 | 39 | 76 |
| 2017 | 17 | 9 | 49 | 75 |
| 2004–2017 | 391 | 145 | 432 | 968 |

Abbreviations: PD, peritoneal dialysis; HD, hemodialysis; (n.), patients number.

# Supplemental Table 2

**Supplemental Table 2.** Detailed characteristics of the study population.

| | Dialysis modality | | | | Missing values (%) | Used as imputation predictor |
|---|---|---|---|---|---|---|
| | CAPD | APD | Home HD | IC-HD | | |
| Number of patients | 162 | 229 | 145 | 432 | 0.0 | No |
| Male (%) | 70 | 66 | 68 | 64 | 0.0 | Yes |
| Number of deaths in 5 years | 67 | 28 | 12 | 174 | 0.0 | No |
| Primary kidney disease (%) [a, b, c] | | | | | 0.0 | Yes |
| Glomerulonephritis | 17 | 22 | 24 | 8 | | |
| Polycystic kidney disease | 6 | 15 | 31 | 8 | | |
| Diabetes | 39 | 30 | 22 | 33 | | |
| Others | 39 | 33 | 29 | 51 | | |
| Cardiovascular comorbid conditions before start of dialysis (%) | | | | | | |
| Acute myocardial infarction | 11 | 5 | 6 | 13 | 0.5 | No |
| Coronary intervention (CABG/PCI) | 11 | 7.4 | 5.5 | 13 | 0.4 | No |
| Angina pectoris / CAD† | 12 | 8 | 5 | 16 | 5.4 | No |
| Coronary angiography | 16 | 5.7 | 6.2 | 13 | 0.4 | No |
| CVA (ischemic) | 1.2 | 0.4 | 1.4 | 1.4 | 0.4 | No |
| CVA (bleeding) | 0.6 | 0.0 | 0.0 | 0.9 | 0.8 | No |
| Peripheral vascular disease (PVD)† | 6.8 | 3.5 | 2.8 | 11 | 0.7 | No |
| PVD with surgical intervention | 6 | 1 | 4 | 10 | 4.2 | No |
| Amputation due to PVD | 1.9 | 1.3 | 0.7 | 5.8 | 3.7 | No |
| Congestive heart failure [c] | 6.8 | 3.1 | 0.7 | 12 | 6.7 | No |
| LVH | 33 | 21 | 17 | 31 | 10 | No |
| Pulmonary embolism | 9.9 | 4.8 | 3.4 | 10 | 0.6 | No |
| Deep vein thrombosis | 2.5 | 0.0 | 2.1 | 1.2 | 1.2 | No |
| Antithrombotic treatment [a, b] | 96 | 93 | 79 | 83 | 2.7 | No |
| Non-Sinus rhythm [b] | 17 | 13 | 4.8 | 43 | 17 | No |
| Irregular rhythm [a, b, c] | 19 | 13 | 6.2 | 78 | 17 | No |
| LVH on EKG [a] | 3.1 | 4.8 | 6.9 | 7.4 | 17 | No |
| LBBB on EKG [a, b, c] | 3.1 | 1.7 | 2.1 | 2.8 | 17 | No |
| Continuous variables (median) | | | | | | |
| Age (years) [a, c] | 65 | 50 | 50 | 66 | 0.0 | Yes |
| Echocardiography | | | | | | |
| Ejection fraction | 60 | 62 | 65 | 60 | 24 | Yes |
| LVEDD (mm) | 49 | 50 | 52 | 51 | 26 | Yes |
| LVESD (mm) [a] | 32 | 32 | 32 | 33 | 48 | |
| LVPW (mm) | 11 | 11 | 11 | 11 | 40 | Yes |
| Septum (mm) | 12 | 12 | 12 | 13 | 38 | Yes |
| Electrocardiogram | | | | | | |
| Heart rate (bpm) | 67 | 67 | 68 | 71 | 17 | Yes |
| PR-interval (ms) | 176 | 162 | 161 | 168 | 25 | Yes |
| QRS-duration (ms) | 94 | 96 | 94 | 96 | 17 | Yes |
| QT-interval (ms) | 416 | 412 | 404 | 414 | 17 | Yes |
| Corrected QT-interval (ms)† | 435 | 435 | 430 | 453 | 17 | Yes |
| P-Axis (degree) [a] | 51 | 53 | 51 | 51 | 26 | Yes |

| | | | | | | |
|---|---|---|---|---|---|---|
| R-Axis (degree) | 17 | 22 | 19 | 17 | 18 | Yes |
| T-Axis (degree) | 54 | 49 | 48 | 58 | 18 | Yes |
| Laboratory findings at start of dialysis | | | | | | |
| P-Creatinine (µmol/l) [a, b] | 557 | 582 | 621 | 556 | 0.6 | Yes |
| P-Urea (mmol/l) | 26 | 27 | 28 | 25 | 1.0 | Yes |
| P-Albumin (g/l) [a, b, c, †] | 36 | 36 | 36 | 30 | 3.9 | Yes |
| P-ionized calcium (mmol/l) [a] | 1.2 | 1.2 | 1.2 | 1.2 | 1.7 | Yes |
| P-Phosphate (mmol/l) | 1.7 | 1.7 | 1.8 | 1.7 | 2.6 | Yes |
| Hemoglobin (g/l) [a, b, c, †] | 114 | 111 | 109 | 100 | 1.5 | Yes |
| P-C-reactive protein (mg/l) [c] | 5 | 4 | 4 | 8 | 5.3 | Yes |
| Total P-Cholesterol (mmol/l) | 4.0 | 4.2 | 4.0 | 3.7 | 16 | Yes |
| High density lipoprotein (mmol/l) | 1.3 | 1.2 | 1.2 | 1.1 | 15 | Yes |
| P-Triglycerides (mmol/l) [b] | 1.6 | 1.4 | 1.4 | 1.4 | 15 | Yes |
| Hemoglobin A1C (mmol/l) | 7 | 7 | 6 | 6 | 53 | No |
| Systolic BP (mmHg) [b, †] | 149 | 144 | 148 | 147 | 4.8 | Yes |
| Diastolic BP (mmHg) [b, †] | 84 | 88 | 84 | 79 | 4.8 | Yes |
| Height (cm) | 170 | 173 | 174 | 171 | 5.0 | Yes |
| Weight (kg) [c] | 75 | 77 | 80 | 77 | 3.0 | Yes |
| Body mass index (kg/$m^2$) [a] | 26 | 25 | 26 | 26 | 6.3 | Yes |

Abbreviations: PD, peritoneal dialysis; CAPD, continuous ambulatory PD; APD, automated PD; HD, hemodialysis; IC-HD, in-center HD; (%), (percentage); CABG, coronary artery bypass grafting; PCI, percutaneous coronary intervention; CAD, coronary artery disease; PVD, peripheral vascular disease; CVA, cerebrovascular accident; LVH, left ventricular hypertrophy; EKG, electrocardiogram; LBBB, left bundle branch block; LVEDD, left ventricular end diastolic diameter; LVESD, left ventricular end systolic diameter; LVPW, left ventricular posterior wall; BP, blood pressure. Characteristics used in calculation of propensity scores, a: CAPD vs IC-HD, b: APD vs IC-HD, c: home HD vs IC-HD. [†]: Possible prognostic factor for major adverse cardiovascular events (MACE).

# Supplemental Table 3

**Supplemental Table 3.** Distribution of MACE according to Dialysis modality

| MACE | CAPD | APD | Home HD | IC-HD | Total |
|---|---|---|---|---|---|
| Cardiac cause of death | 21 | 9 | 5 | 66 | 101 |
| Myocardial infarction | 9 | 10 | 4 | 30 | 53 |
| Ischemic stroke | 10 | 6 | 1 | 15 | 32 |
| Hemorrhagic stroke | 1 | 1 | 1 | 4 | 7 |
| Total | 41 | 26 | 11 | 115 | 193 |

Abbreviations: MACE, major adverse cardiovascular events; PD, peritoneal dialysis; APD, automated PD; CAPD, continuous ambulatory PD; HD, hemodialysis; IC-HD, in-center HD; CVA, cerebrovascular accident; (n), number.

# Supplemental Table 4

**Supplemental Table 4.** Comparison of hazard ratios of MACE between males and females with propensity score adjustment

| Comparison | HR (95% CI) males | HR (95% CI) females | P value for interaction |
|---|---|---|---|
| Compared to in-center HD | | | |
| PD | 1.80 (1.11–2.92) | 0.37 (0.14–0.99) | 0.006 |
| CAPD | 1.78 (1.02–3.09) | 0.25 (0.07–0.93) | 0.04 |
| APD | 1.26 (0.65–2.45) | 0.22 (0.05–0.94) | 0.06 |
| Home HD | 0.62 (0.21–1.80) | 0.73 (0.20–2.57) | 0.33 |
| Compared to home HD | | | |
| PD | 2.06 (0.82–5.21) | 0.32 (0.09–1.17) | 0.01 |
| CAPD | 2.09 (0.72–6.08) | 0.09 (0.01–0.79) | 0.01 |
| APD | 1.73 (0.59–5.01) | 0.33 (0.07–1.58) | 0.02 |

Abbreviations: MACE, major adverse cardiovascular events; KRT, kidney replacement therapy; HR, hazard ratio; CI, confidence interval; PD, peritoneal dialysis; HD, hemodialysis; CAPD, continuous ambulatory PD; APD, automated PD.

# Supplemental Table 5

**Supplemental Table 5.** Hazard ratios of the first MACE according to dialysis modality during 5 years from the start of KRT, with censoring at change of dialysis modality, kidney transplantation, loss of follow-up, recovery of kidney function, non-cardiac death, and end of follow-up.

| | HR of first MACE | 95% CI of HR | |
|---|---|---|---|
| | | Lower | Upper |
| HR of first MACE, unadjusted | | | |
| IC-HD (reference) | | | |
| CAPD | 0.64 | 0.40 | 1.02 |
| APD | 0.51 | 0.31 | 0.84 |
| Home HD | 0.43 | 0.23 | 0.83 |
| HR of first MACE, adjusted for age and sex | | | |
| IC-HD (reference) | | | |
| CAPD | 0.64 | 0.40 | 1.03 |
| APD | 0.66 | 0.39 | 1.10 |
| Home HD | 0.57 | 0.29 | 1.12 |
| HR of first MACE, adjusted for propensity score | | | |
| CAPD (compared to IC-HD) | 1.06 | 0.56 | 1.98 |
| APD (compared to IC-HD) | 1.23 | 0.62 | 2.41 |
| Home HD (compared to IC-HD) | 0.93 | 0.37 | 2.35 |

Abbreviations: MACE, major adverse cardiovascular events; KRT, kidney replacement therapy; HR, hazard ratio; CI, confidence interval; HD, hemodialysis; PD, peritoneal dialysis; APD, automated PD; CAPD, continuous ambulatory PD; IC-HD, in-center HD.
